# Place of death among older people in Finland and Norway

**DOI:** 10.1177/1403494820944073

**Published:** 2020-08-05

**Authors:** Leena Forma, Mari Aaltonen, Jani Raitanen, Kjartan S. Anthun, Jorid Kalseth

**Affiliations:** 1 Faculty of Social Sciences (Health Sciences) and Gerontology Research Centre (GEREC), Tampere University, Finland; 2 Faculty of Social Sciences, University of Helsinki, Finland; 3 UKK Institute for Health Promotion Research, Finland; 4 Department of Health Research, SINTEF Digital, Norway

**Keywords:** Place of death, older people, end-of-life trajectory, register study, hospital, long-term care, Finland, Norway

## Abstract

**Aims:** This study aimed to find out how place of death varied between countries with different health and social service systems. This was done by investigating typical groups (concerning age, sex and end-of-life trajectory) of older people dying in different places in Finland and Norway. **Methods:** The data were derived from national registers. All those who died in Finland or Norway at the age of ⩾70 years in 2011 were included. Place of death was analysed by age, sex, end-of-life trajectory and degree of urbanisation of the municipality of residence. Two-proportion *z*-tests were performed to test the differences between the countries. Multinomial logistic regression analyses were performed separately for both countries to find the factors associated with place of death. **Results:** The data consisted of 68,433 individuals. Deaths occurred most commonly in health centres in Finland and in nursing homes in Norway. Deaths in hospital were more common in Norway than they were in Finland. In both countries, deaths in hospital were more common among younger people and men. Deaths in nursing homes were commonest among frail older people, while most of those who had a terminal illness died in health centres in Finland and in nursing homes in Norway. ***Conclusions:* Both Finland and Norway have a relatively low share of hospital deaths among older people. Both countries have developed alternatives to end-of-life care in hospital, allowing for spending the last days or weeks of life closer to home. In Finland, health centres play a key role in end-of-life care, while in Norway nursing homes serve this role.**

## Introduction

Death is being delayed to an increasingly older age, and therefore the role of long-term care (LTC) facilities as the place of death is expected to rise. Death in residential care is more common among much older people in, for example, Finland [1], Norway [2], Sweden [3] and a comparison of 36 countries [4]. In addition, increasing longevity is likely to increase the number of people with dementia. Most severely cognitively impaired people often die in residential care [5–7].

Place of death is associated with the health and social service system, for example the availability of hospital and nursing-home beds [5,8–11], home-care resources [12] or palliative care at home or in care settings [13]. We aimed to understand which groups of older people die in different types of institutions in Finland and Norway. We applied the Andersen model on health-care use [14]. Underlying determinants of service use (here, place of death) are individual predisposing, enabling and need factors. In addition to individual factors, service use takes place in certain environments, where the local health and social service system provides the services, determined by the health and social policy. In present study, we take into account individual factors and hence try to find variation which is assumed to be associated with the service system.

The main motivation for comparing Finland and Norway is that although these two countries are similar in many ways, there are also remarkable differences in the way services for older people are organised. The population sizes are quite similar (>5 million) in Finland and Norway, and they both have well-developed welfare states, with mainly publicly organised and funded health and LTC services. In both countries, responsibility for organising LTC and primary care for residents lies with municipalities. In Finland, municipalities are also responsible for providing hospital services, although these are organised within hospital districts comprising several municipalities. Norway’s secondary health-care services are state owned, with regional health authorities responsible for provision; the services are organised in hospital trusts with geographical catchment areas. The funding of services is tax based but requires co-payments from the users of some services in both countries [15,16].

Care for older people is provided mainly in private homes as informal and formal care and in round-the-clock care facilities such as nursing homes and sheltered housing. In addition, older people are frequent attenders for hospital care, and therefore we provide here background information for that too. The availability of somatic and psychiatric hospital beds is higher in Norway than in Finland, but in Finland there are many more ‘other’ hospital beds, and Finland has more hospital beds in total (Table I). In practice, all hospitals in the ‘other’ category in Finland are primary-care hospitals (hereafter health centres [17]), some of which are similar to nursing homes in other Nordic countries [18]. They provide both acute care and LTC.

**Table I.** Population and health statistics for Finland and Norway.

	Finland	Norway
Population aged 65+ (%)^a^	17.3	15.0
Population aged 80+ (%)^a^	4.7	4.5
Life expectancy at age 65 (years)^a^	19.9	20.0
Healthy life years at age 65 (years)^a^		
Women	8	15
Men	7	15
Limitations in daily activities in adults aged 65+ (%)^a^	53.9	23.4
Strong limitations in daily activities in adults aged 65+ (%)^a^	15.0	10.1
Estimated prevalence of dementia (*n* per 1000 population)^a^	18.2	15.4
Population receiving long-term care aged 65–79 (%)^a^	0.7	0.8
Population receiving long-term care aged 80+ (%)^a^	1.5	1.8
Long-term care recipients aged 65+ receiving care at home (%)^a^	60.0	70.8
Somatic hospital beds (*n* per 100,000 population)^b^	172	288
Psychiatric hospital beds (*n* per 100,000 population)^b^	71	91
Other hospital beds (*n* per 100,000 population)^b^	312	38
Hospital beds in total (*n* per 100,000 population)^b^	555	417
Long-term care beds in institutions (*n* per 1000 population aged 65+)^a^	60.5	54.3
Long-term care beds in hospitals (*n* per 1000 population aged 65+)^a^	6.9	
Long-term care public expenditure (health and social components; % of GDP)^a^	2.2	2.4
Long-term care recipients aged 65+ in institutions (other than hospitals; % of the population aged 65+)^c^	5.0	5.3
Long-term care recipients age 8 0+ in institutions (other than hospitals; % of the population aged 80+)^c^	13.3	14.1
Long-term care recipients aged 65+ at home (% of the population aged 65+)^c^	7.5	12.4
Long-term care recipients aged 80+ at home (% of the population aged 80+)^c^	19.0	28.1
People living at institutions or in service housing, total aged 65+ (%)^d^	5.1	7.3
People living at institutions or in service housing, aged 65–74 (%)^d^	1.5	2.0
People living at institutions or in service housing, aged 75–79 (%)^d^	3.7	5.1
People living at institutions or in service housing, aged 80+ (%)^d^	14.2	20.8

a Health at a Glance 2015: OECD Indicators (most indicators from 2013 or nearest).

b Nomesko. Health statistics in the Nordic countries 2013. Report no. 100:2013. Copenhagen: Nordic Medico-Statistical Committee.

c OECD stat indicators from 2011.

d Nomesko. Health and health care of the elderly in the Nordic Countries: From a statistical perspective. 2017. Data for Finland from 2015 and for Norway 2016.

The number of beds in residential care facilities per 1000 inhabitants aged ⩾65 years was higher in Finland than in Norway in 2011 (Table I). The share of LTC users (institutional and home care, excluding health centres) was found to be higher in Norway than in Finland. In particular, the use of home care is higher in Norway than in Finland [19]. In both countries, nursing homes provide both short-term and LTC stays. In summary, the main differences in care for older people between the countries are (a) the use of health centres in Finland and (b) the higher share of LTC, particularly home care, in Norway. The role of health centres in Finland compared to places of death in Norway is unknown. Do health centres play the role of hospital beds or nursing home beds in Norway, and how does this role vary according to age, sex and end-of-life trajectory?

### Aims

The aim of this study was to explore how place of death varied between countries with different health and social service systems. This was done by investigating typical groups of older people dying in different places in Finland and Norway. In addition, we provide information on the transitions at the end of life: from where the person came to the place of death and how long he/she stayed there. The more detailed research questions were:

(1) What were the places of death of women and men aged 70–74, 75–79, 80–84, 85–89, 90–94 and 95+ in Finland and Norway?
(2) How was end-of-life trajectory associated with place of death among older people in Finland and Norway?

The study is part of more comprehensive projects: New Dynamic of Longevity and the Changing Needs for Services (COCTEL; Finland) and Utilisation of Healthcare Services at the End of Life (Norway).

## Methods

### Data

The study population consisted of all those who died at the age of ⩾70 years in Finland or Norway in 2011. People were identified from the Finnish Causes of Death Register (Statistics Finland) and the Norwegian Cause of Death Registry (Norwegian Institute of Public Health). Data on place of death were derived from the Care Register for Healthcare and the Care Register for Social Welfare (National Institute for Health and Welfare) for Finland, and from the Cause of Death Registry and Patient Registry (Norwegian Directorate of Health) for Norway. In both countries, the data from different registers were linked using personal identification codes. These were replaced with research numbers before the data were given to the researchers. More detailed descriptions of the data collection have been given elsewhere [2,20,21].

To describe transitions to and the number of days in place of death, a second data set for Norway is used including, in addition to Cause of Death Registry data and data from Norwegian Patient Registry, data on out-of-hospital institutional care, that is, short- and long-term nursing-home care, and place of residence (long-term nursing home, sheltered housing and ordinary home) on the day of death. These data were obtained from the Norwegian Information System for the Nursing and Care Sector (IPLOS database; for details, see Table IV).

### Variables

The dependent variable is place of death, defined in Table II. For Finland, place of death was identified by the last place where the person was recorded on the day of death in the care registers. If the person was not in a care facility on the day of death, she/he was considered to have died not in an institution (e.g. at home). For Norway, place of death was derived from the Cause of Death Registry. The categories ‘hospital’, ‘nursing home’ and ‘sheltered housing’ are quite clear. Primary- and secondary-care hospitals could have been combined as a single hospital category, but our aim was to differentiate the role of the health centre (primary-care hospital).

**Table II.** Definitions of place of death (PoD) and data sources.

PoD	Finland	Data source	Norway	Data source
Hospital	Secondary care: university, central, regional and private hospitals	CRHC	Somatic and psychiatric hospitals	NCDR
Health centre	Primary-care hospital	CRHC		
Nursing home	Residential home (social care)	CRSW	Nursing homes and other LTC institutional facilities	NCDR
Sheltered housing	Sheltered housing with 24-hour assistance (social care)	CRSW	Sheltered housing (possibly including less than 24-hour assistance)	IPLOS
Not in institution	Private home, transport (death abroad excluded)	CRSW, or not in care registers on day of death	All other (home, transport, outdoors etc.; death abroad excluded)	NCDR

CRHC: Care Register for Healthcare; CRSW: Care Register for Social Welfare; NCDR: Norwegian Cause of Death Registry; IPLOS: Norwegian Information System for the Nursing and Care Sector.

The independent variables are age at death (70–74, 75–79, 80–84, 85–89, 90–94 and 95+), sex (predisposing factors), end-of-life trajectory (need factor) and degree of urbanisation of the municipality of residence (enabling factor).

Instead of separate causes of death, we studied end-of-life trajectories. The trajectories have been developed based on functional decline and resource utilisation at the end of life, and they reflect how function is decreasing: whether it is high and then falls rapidly or it is low for a longer period and smoothly or fluctuating decreases towards death. The classification of end-of-life trajectories (sudden death, terminal illness, organ failure, frailty and other) is based on underlying causes of death such as in Fassbender et al. [22] and the Canadian Institute for Health Information [23].

The degree of urbanisation was considered to reflect the distance to hospital. Municipalities were defined as cities (densely populated areas), towns and suburbs (intermediate density areas) or rural areas (sparsely populated areas) [24].

### Statistical analyses

We performed parallel analyses with the Finnish and Norwegian data sets. To test the differences between the countries, we performed two-proportion *z*-tests for places of death. This was done separately for age groups, sex and end-of-life trajectories. Multinomial logistic regression analyses were performed separately for Finland and Norway to find the associations of age, sex, end-of-life trajectory and degree of urbanisation with place of death. People living in the same municipality were clustered because their place of death was assumed to be correlated, as service supply differs between the municipalities.

The analyses were performed with Stata v15.0 (StataCorp, College Station, TX) and IBM SPSS Statistics for Windows v25 (IBM Corp., Armonk, NY). The Pirkanmaa Hospital District Ethics Committee approved the COCTEL study plan; the Regional Committee for Medical and Health Research Ethics for Central Norway (approval no. 2012/852) and Norwegian Social Science Data Services (Privacy Ombudsman for Research) approved the Norwegian study plan.

## Results

### Descriptives

In 2011, 37,051 people in Finland and 31,382 in Norway died at the age of ⩾70 years (Table III). The Finnish decedents were younger (*M*_age_=83.6 years) than the Norwegian decedents (*M*_age_=84.9 years). The commonest end-of-life trajectories were frailty and organ failure in both countries (46% and 28% in Finland and 31% and 37% in Norway, respectively; Table III).

**Table III.** Descriptives.

	Finland	Norway	*p*-Value
Total number of deaths	37,051	31,382	
PoD, %			<0.001
Hospital	20.5	31.9	
Health centre	45.6		
Nursing home	11.8	54.9^a^	
Sheltered housing	7.2	2.4	
Not in institution	15.0	10.8	
End-of-life trajectories, %			<0.001
Sudden death	1.2	4.1	
Terminal illness	21.2	23.8	
Organ failure	28.1	36.9	
Frailty	46.0	31.0	
Other	3.5	4.2	
Women, %	56.5	55.7	<0.05
Age (years), %			<0.001
70–74	13.5	9.8	
75–79	16.2	14.2	
80–84	23.4	21.3	
85–89	24.7	26.7	
90–94	15.8	20.1	
95+	6.4	7.9	
Degree of urbanisation of municipalities (of study population living in), %			<0.001
City	28.9	22.7	
Town or suburb	27.5	32.7	
Rural area	43.7	44.5	

*p*-Values refer to two-proportion *z*-tests.

a Based on information from IPLOS, we estimate that about 35% of all deaths in institutional care outside hospital are temporary stays, and about 65% are long-term stay (permanent resident) in a nursing home.

**Table IV.** Number of days in the PoD and the last place before PoD.

	Finland					Norway^a^					
	Hospital	Health centre	Nursing home	Sheltered housing	Not in institution	Hospital	Nursing home	Short term	Long term	Sheltered housing	Not in institution
Days in PoD											
*M* (*SD*)	15 (44)	69 (110)	233 (149)	196 (151)	214 (156)	10 (140)	166 (155)	25 (33)	212 (153)	190 (153)	190 (162)
Median	5	20	347	183	277	6	92	15	247	161	170
Last place before PoD (%)											
No transfers^b^	1.1	9.4	48.9	36.5	46.1	0	32.2	0.3	42.8	35.7	40.4
Hospital		41.2	9.2	11.1	20.5		53.3	78.2	33.1	47.8	40.1
Health centre	19.1		30.3	36.9	27.5						
Nursing home	2.7	2.9		2.4	2.9	22.5				10.3	18.3
*Short term*						*11.4*			*17.3*	*9.9*	*11.3*
*Long term*						*11.1*		*0.5*		*0.4*	*7.0*
Sheltered housing	5.2	8.3	2.5		3.0	9.3	2.7	2.8	1.7		1.2
Home	71.9	38.2	9.1	13.1		68.3	11.8	18.3	5.2	6.3	
Total	100	100	100	100	100	100	100	100	100	100	100

a In the data used for Norway, the age limit is set at 71 years due to restrictions from the data owner, and hence the number of deaths is lower. Also, 1870 deaths registered with PoD in a LTC institution but not registered with a LTC institutional stay on the day of death in the IPLOS data were excluded.

b No transfers in the last year of life.

### Place of death, age, sex and end-of-life trajectory

Deaths occurred most commonly in health centres in Finland (46%) and in nursing homes in Norway (55%; Table III). Deaths in nursing homes were more common among those who were much older. They were also more common among women than among men (Supplemental Figure S1).

Hospital deaths were more common in Norway than they were in Finland in all categories (age, sex and end-of-life trajectory), apart from people whose death was sudden (Supplemental Figure S1). The proportion of hospital deaths decreased gradually from the youngest to the oldest and was higher among men than women (Supplemental Figure S1).

### The last transition

Of those who died in nursing homes (long-term stay in Norway), sheltered housing or at home, 36–49% did not have any transitions in their last year of life (Table IV). Thus, the median numbers of days in these places of death were high. Most of those who died in hospital went there from home. About one fifth were admitted to hospital from a health centre in Finland and from a nursing home in Norway. The opposite also applies: those who died in a health centre in Finland and a nursing home (especially short-term stay) in Norway most frequently came from hospital. Another common path in end-of-life care was from a health centre to a nursing home in Finland and from a short-term nursing home stay to a long-term placement in Norway.

### Multivariate analyses

Older and women were more likely than younger people and men to die in health centres or nursing homes than in hospitals (Table V). The pattern was similar for ‘not in institution’ for the two oldest age groups and for women in Finland. The 80–94 age groups were less likely to die ‘not in institution’ than the youngest in Norway.

**Table V.** Relative risk ratios of factors associated with PoD.

	Health centre	Nursing home		Not in institution	
	Fin	Fin	No	Fin	No
Age group (ref. 70–74)					
75–79	1.25***	1.68***	1.52***	0.91	0.88*
80–84	1.62***	2.24***	2.06***	0.86**	0.75***
85–89	2.08***	3.13***	3.10***	0.95	0.75***
90–94	2.83***	5.46***	4.53***	1.24**	0.79**
95+	3.76***	9.26***	9.54***	1.77***	1.17
Sex (ref. woman)	0.78***	0.56***	0.69***	0.90**	1.05
End-of-life trajectory (ref. terminal illness)					
Sudden death	0.24***	1.15	0.91	14.8***	5.02***
Organ failure	0.62***	1.31*	0.64***	2.02***	1.33***
Frailty	0.94	5.08***	1.09**	5.00***	1.94***
Other	0.43***	0.60**	0.44***	1.14	1.03
Urbanisation (ref. rural area)					
City	0.75	1.26	0.78***	0.99	0.60***
Town or suburb	0.84	0.84	0.83***	0.93	0.77***
Constant	2.07***	0.10***	1.06	0.43***	0.41***

Multinomial regression analyses for Finland (Fin) and Norway (No) (ref. hospital). People living in the same municipality are clustered.

In the Norwegian data, 214 deaths registered as death in hospital in the Cause of Death Registry was not registered in hospital at day of death in the data from Norwegian Patient Registry. These were excluded from the regression.

* *p*<0.05; **p<0.01; ***p<0.001.

In these analyses, the reference category for end-of-life trajectory was terminal illness. Those whose trajectory was sudden death were much more likely to die ‘not in institution’ than in hospital (Table V). Those whose trajectory was organ failure were more likely to die in hospital than in a health centre or nursing home in Norway, but were more likely to die ‘not in institution’ in Finland. Those whose trajectory was frailty were less likely to die in hospital than in other places, but there was no statistically significant difference for health centres.

There were differences between urban and rural municipalities in Norway but not in Finland (Table V). In Norway, those who lived in rural municipalities were less likely to die in hospital than those living in cities, towns or suburbs.

## Discussion

The purpose of the study was to find out how place of death of older people varies between Finland and Norway, where ways to organise services differ. There were similarities between the countries: a higher share of much older people and women died in nursing homes, while a higher share of younger people and men died in hospitals.

However, the roles of hospitals and nursing homes as places of death differed between the countries. Death in hospital was more common in Norway than in Finland. In Finland, health centres were the commonest place of death, while in Norway it was nursing homes.

There are some similarities between health centres in Finland and nursing homes (especially short-term nursing homes) in Norway: people have transitions from these to hospital but more often from hospital to these facilities before death. The study also confirms the importance of nursing homes as places of end-of-life care in Norway, especially for those with terminal illnesses [2]. Nursing homes provide not only long-term residence for the frailest older people, but also short-term stays for patients who no longer need specialised hospital treatment. Nearly 40% of those who died in nursing homes in Norway in our data were not long-term residents on the day of death (data not shown). This suggests that nursing homes in Norway to some degree play the same role as health centres in Finland. This trend is likely to have been reinforced by the coordination reform introduced in Norway in 2012. One important goal of the reform was to enable and encourage municipalities to prevent avoidable hospital admissions, and to take care of patients who were ready for discharge from hospital. One means to this end was to make it mandatory for municipalities to provide acute beds.

We compared place of death and took into account individual predisposing, enabling and need factors like the Andersen model presumes [14]. There were differences between these individual factors between the countries, especially in end-of-life trajectories. But there was also variation in the way individual factors were associated with the place of death, for example there was a difference between people living in rural areas compared to those living in towns, suburbs or cities in Norway but not in Finland.

In several countries, a remarkable share of older people (from 35% in New Zealand to nearly 70% in Japan) die in hospital [4]. Internationally, hospital is the commonest place of death among older people. In Sweden, another Nordic welfare state, the share is 40% [3]. Therefore, both Finland and Norway differ from other countries in having lower shares of hospital deaths. This is likely related to the role of health centres in Finland and the ‘extended’ role of nursing homes as places of end-of-life care in Norway.

Only 7% of deaths of older people are classified as sudden [25]. Therefore, it is possible to plan end-of-life care to some extent. People dying of different chronic conditions have different end-of-life trajectories and therefore different pathways of care, including place of death [25]. The place of death reflects the place of living near the time of death and an earlier use of services. End-of-life trajectories that are more likely to follow predictable care patterns, such as those in dementia [26], will help the planning and allocation of care in advance.

In Finland, care in health centres has been between hospital and nursing-home care and has served a variety of people living their last stages of life with terminal illness, frailty or organ failure. The actual LTC facilities (i.e. nursing homes and sheltered housing) seem to be targeted at people whose trajectory is frailty. In Norway, end-of-life care for people with different trajectories is more evenly distributed between the care sites.

Even though the transitions between care facilities during the last year of life are common, many spent the whole last year of life in the same place in both countries. When the health status requires, transitions are needed, but there is also the possibility of avoidable transitions. This question is especially relevant when moving from an LTC facility to a hospital. Could care be offered on-site? Avoidable transitions can burden both the care system and the care recipient, in particularly those who are frail.

### Strengths and limitations

Including whole populations who died at the age of ⩾70 years and using comprehensive register data are strengths of this study.

There may be differences between the countries’ definitions of underlying cause of death and thus also in the end-of-life trajectories. Frailty was the end-of-life trajectory for 46% of the study population in Finland, and for 31% in Norway. It is unlikely that there is a remarkable difference between the countries in the prevalence of infections, dementia, chronic heart disease and other causes underlying this category. Multi-morbidity is common among frail older people, which complicates the classification of underlying causes of death [27], and this may hamper the comparability of causes of death between the countries.

There are also some differences between the countries’ definitions of place of death. In the Finnish data, the definition was made on the basis of care registers, but in the Norwegian data it was mainly on the basis of the Cause of Death Registry.

Analyses with pooled Finnish and Norwegian data sets would have allowed controls for variations in, for example, age and end-of-life trajectory, and thus strengthened our results. However, we had no permission for pooling.

The ‘not in institution’ place of death category includes both sheltered housing and home, as well as transport, death outside and missing place of death. Thus, it is a very heterogeneous group. Sheltered housing with 24-hour assistance is an increasingly common place of death in Finland [28]. A very high proportion (76%) of those dying of dementia in ‘other’ places died in sheltered housing.

Further research questions arise based on the current study. Since 2011, some changes have occurred. In Norway, municipal acute care units were implemented in 2012. These are intermediate units that aim to reduce hospital admissions, especially among older people [29]. This may have reinforced the trend towards local institutional care as the place of death among older people. In Finland, the role of health centres as the place of death among older people has decreased, and deaths in nursing homes and sheltered housing with 24-hour assistance have become more common [28].

It would be important to study the quality of end-of-life care and the quality of death. Place of living is an important factor defining use of care and place of death [30], and it should be included in further analyses. It would also be important to include other Nordic countries in the comparison.

## Conclusions

Both countries have developed alternatives to end-of-life care in hospital, allowing for spending the last days or weeks of life and dying closer to home. In Finland, health centres play a key role in end-of-life care and place of death, while in Norway nursing homes serve this role.

## Supplemental Material

SupplementaryFigure1 – Supplemental material for Place of death among older people in Finland and Norway Click here for additional data file. Supplemental material, SupplementaryFigure1 for Place of death among older people in Finland and Norway by Leena Forma, Mari Aaltonen, Jani Raitanen, Kjartan S. Anthun and Jorid Kalseth in Scandinavian Journal of Public Health
