# Supplementary figures and images for: Place of death among older people in Finland and Norway
Source: Scand J Public Health. 2020 Aug 5;48(8):817–24. doi: 10.1177/1403494820944073 (PMC7678340; doi:10.1177/1403494820944073)

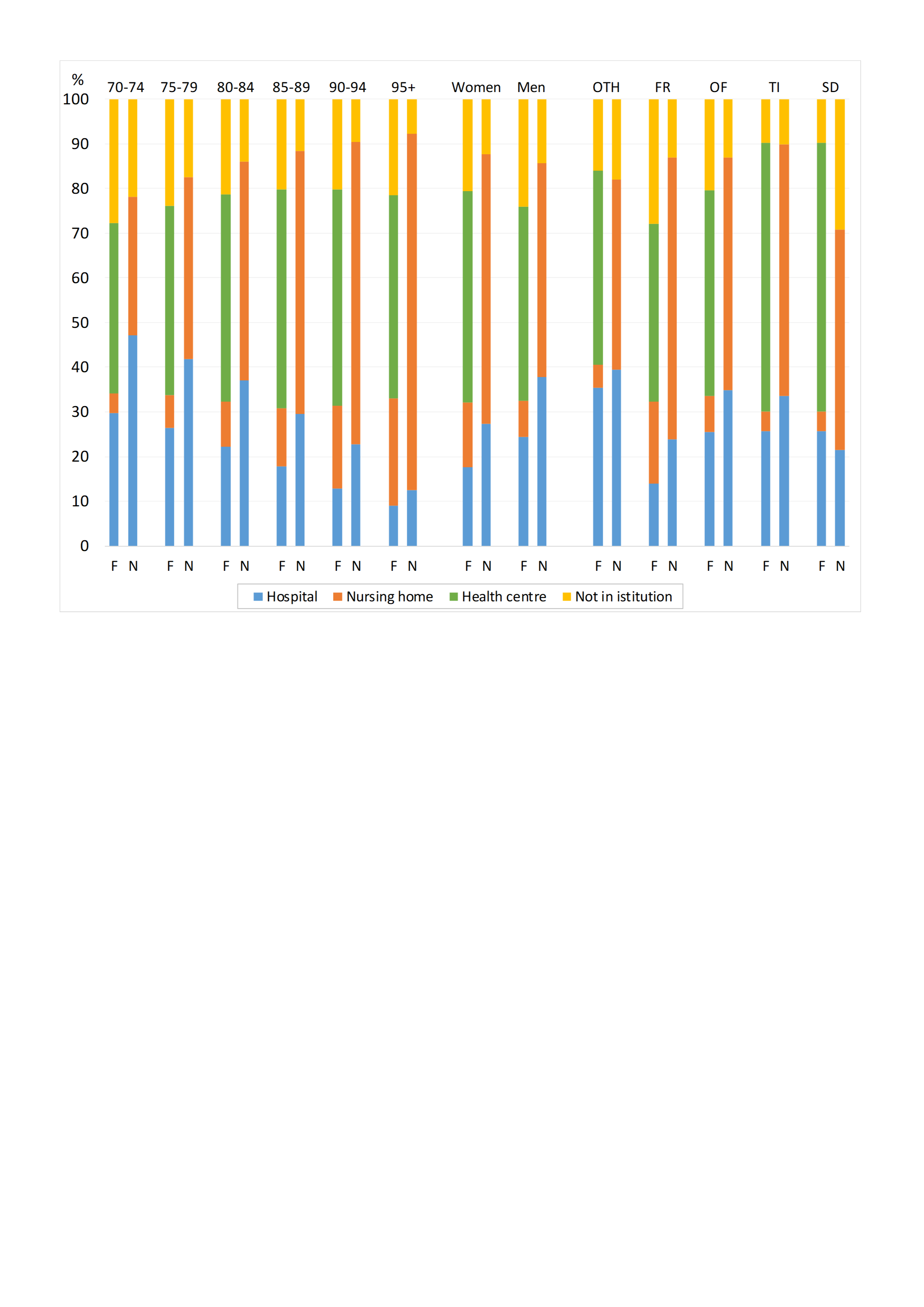

Supplement: SupplementaryFigure1 – Supplemental material for Place of death among older people in Finland and Norway [file SupplementaryFigure1.tif]
